# P-1085. Characterization of *Enterobacter cloacae* and *Citrobacter freundii* Species Ccomplex Isolates with Decreased Susceptibility to Cephalosporins from United States Hospitals and Activity of Aztreonam-avibactam and Comparator Agents (2019-2023)

**DOI:** 10.1093/ofid/ofae631.1273

**Published:** 2025-01-29

**Authors:** Helio S Sader, Timothy Doyle, Hank Kimbrough, Cory Hubler, Mariana Castanheira

**Affiliations:** JMI Laboratories, North Liberty, Iowa; Element Materials Technology/Jones Microbiology Institute, North Liberty, Iowa; Element Materials Technology/Jones Microbiology Institute, North Liberty, Iowa; Element Materials Technology/Jones Microbiology Institute, North Liberty, Iowa; JMI Laboratories, North Liberty, Iowa

## Abstract

**Background:**

Aztreonam-avibactam (ATM-AVI) is under clinical development for treatment of Gram-negative infections, including those caused by metallo-β-lactamase (MBL) producers. We evaluated the activity of ATM-AVI and comparators against cephalosporin non-susceptible (S) *E. cloacae* (ECLC) and *C. freundii* species complex (CFC) from patients hospitalized in United States (US) medical centers.

Antimicrobial susceptibility of cephalosporin-non-susceptible E. cloacae and C. freundii species complex

**Figure:**
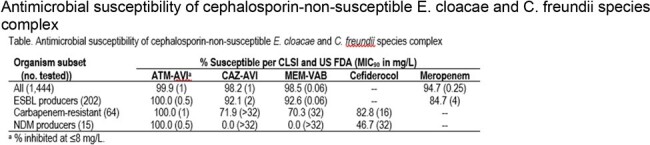

**Methods:**

A total of 43,235 Enterobacterales (1/patient) were collected via the INFORM Surveillance Program and susceptibility tested by broth microdilution in 2019-2023; among those, 5,106 (11.8%) were ECLC (n=3,732) or CFC (n=1,374). We evaluated the antimicrobial susceptibility and β-lactamase production of isolates ceftazidime-resistant (MIC ≥ 16 mg/L) or cefepime-non-S (MIC ≥ 2 mg/L). The collection includes 1,065 ECLC and 379 CFC. ATM-AVI was tested with AVI at fixed 4 mg/L and a PK/PD S breakpoint of ≤ 8 mg/L was applied for comparison. Comparator included ceftazidime-avibactam (CAZ-AVI), meropenem-vaborbactam (MEM-VAB), and cefiderocol (carbapenem-resistant [CB-R] isolates only), among others. All isolates (n=1,444) were characterized by whole genome sequencing.

**Results:**

Isolates were mainly from urinary tract (35.5%), pneumonia (23.6%), and bloodstream infection (BSI; 16.1%). The most common ESBLs were CTX-M type (99; 49.0% of ESBL producers), SHV type (95; 47.0%), and OXA type (80; 39.6%); ≥ 2 ESBLs were identified in 67 isolates (33.1%), mainly OXA-1/30 plus a CTX-M (64 isolates; 31.7%). A carbapenemase was identified in 55 of 64 (85.9%) CB-R isolates; including KPC type (40 isolates; 62.5% of CB-R) and NDM-1 (15; 23.4% of CB-R). ATM-AVI inhibited 99.9% of isolates at ≤ 8 mg/L and showed complete activity (100.0% inhibited at ≤ 8 mg/L) against ESBL producers and CB-R isolates, including NDM producers (Table). CAZ-AVI and MEM-VAB were active against 92.1-92.6% of ESBL producers and 70.3-71.9% of CB-R isolates. Cefiderocol was active against 83.9% of CB-R isolates but only 46.7% of NDM producers.

**Conclusion:**

ATM-AVI was highly active against cephalosporin-non-S ECLC and CFC, including MBL producers. The activities of CAZ-AVI, MEM-VAB, and cefiderocol were compromised against CB-R isolates due to the high frequency of NDM producers.

**Disclosures:**

**All Authors**: No reported disclosures

